# Dynamic and living devices for overcoming fibrosis of implanted biomaterials

**DOI:** 10.1002/btm2.70048

**Published:** 2025-07-21

**Authors:** Joy M. Jackson, Lolita Testu, Alex Abramson

**Affiliations:** ^1^ The Wallace H. Coulter Department of Biomedical Engineering Georgia Institute of Technology Atlanta Georgia USA; ^2^ School of Chemical and Biomolecular Engineering Georgia Institute of Technology Atlanta Georgia USA; ^3^ Division of Digestive Diseases Emory University School of Medicine Atlanta Georgia USA

**Keywords:** bioelectronics, biomaterials, fibrosis, foreign body response, implants, medical devices

## Abstract

The fibrotic encapsulation of implantable medical devices reduces diffusion‐based mass transport and electrical conductivity between the tissue and implant, limiting many devices to weeks‐long rather than years‐long lifetimes. Most strategies to overcome fibrosis take a passive, materials‐driven approach to mitigate the chemical and mechanical mismatch at the tissue‐implant interface through superficial or structural implant modifications. Recent advancements in microfabrication and mechanotherapy have led engineers to incorporate smart and active mechanical actuation systems into implantable devices that use pressure, vibration, and integrated electronics to perpetually overcome effects of the foreign body response. Here, we highlight medical applications where active antifibrotic strategies outperform passive strategies in terms of device lifetimes and therapeutic outcomes, outline engineering design considerations for integrating active strategies, and discuss challenges in developing dynamic and living implants.

**Translational Impact Statement**

The immune response to implantable medical devices, such as drug delivery systems and biosensors, often compromises their long‐term efficacy. While traditional strategies focus on physical or chemical surface modifications, emerging mechanotherapy and bioelectronic approaches offer programmable, on‐demand interventions to actively modulate the immune response. These innovations have the potential to enhance device performance, extend longevity, and improve patient outcomes by dynamically adapting to the biological environment.

## 1 INTRODUCTION

Implantable medical devices improve patient compliance and health outcomes by offering continuous disease monitoring and treatment; however, the body's immune response limits the functionality and lifespan of these systems. This immune cascade, known as the foreign body response, culminates in fibrotic encapsulation of the implanted biomaterial.^1^ For implantable drug delivery systems, biosensors, and cell encapsulation devices, fibrotic tissue acts as a diffusion barrier^2^, ^3^, ^4^, ^5^ that impedes the passive transport of molecules in time‐sensitive applications. Continuous glucose monitors, for example, rely on rapid diffusion to provide real‐time data that informs insulin dosing, but readings can be delayed up to 20 min after 1–2 weeks of implantation; 80% of this delay can be attributed to fibrotic encapsulation.^6^, ^7^ Implant fibrosis also exhibits high resistivity,^8^ which impairs electronic stimulation devices such as cochlear implants^9^ and pacemakers.^10^ Overall, the foreign body response accounts for 10% of implant failures annually,^11^ driving researchers to engineer targeted mitigation strategies.

Researchers have long utilized passive strategies involving physical and chemical surface modifications to mitigate the foreign body response to implanted devices^11^, ^12^, ^13^; however, recent advances in microfabrication and additive manufacturing have enabled the engineering of implantable robotic devices that actively provide mechanical stimulation to further enhance and extend control over fibrotic encapsulation. While passive strategies aim to reduce the mismatch of chemical and physical properties at the tissue‐implant interface, active strategies leverage mechanotherapy to disrupt cell adhesion and alter cell behavior. Here, we highlight how on‐demand, electrical and mechanical actuation enables long‐term implant efficacy in applications where surface modifications alone provide limited effects.

## 2 THE FOREIGN BODY RESPONSE

**FIGURE 1:**
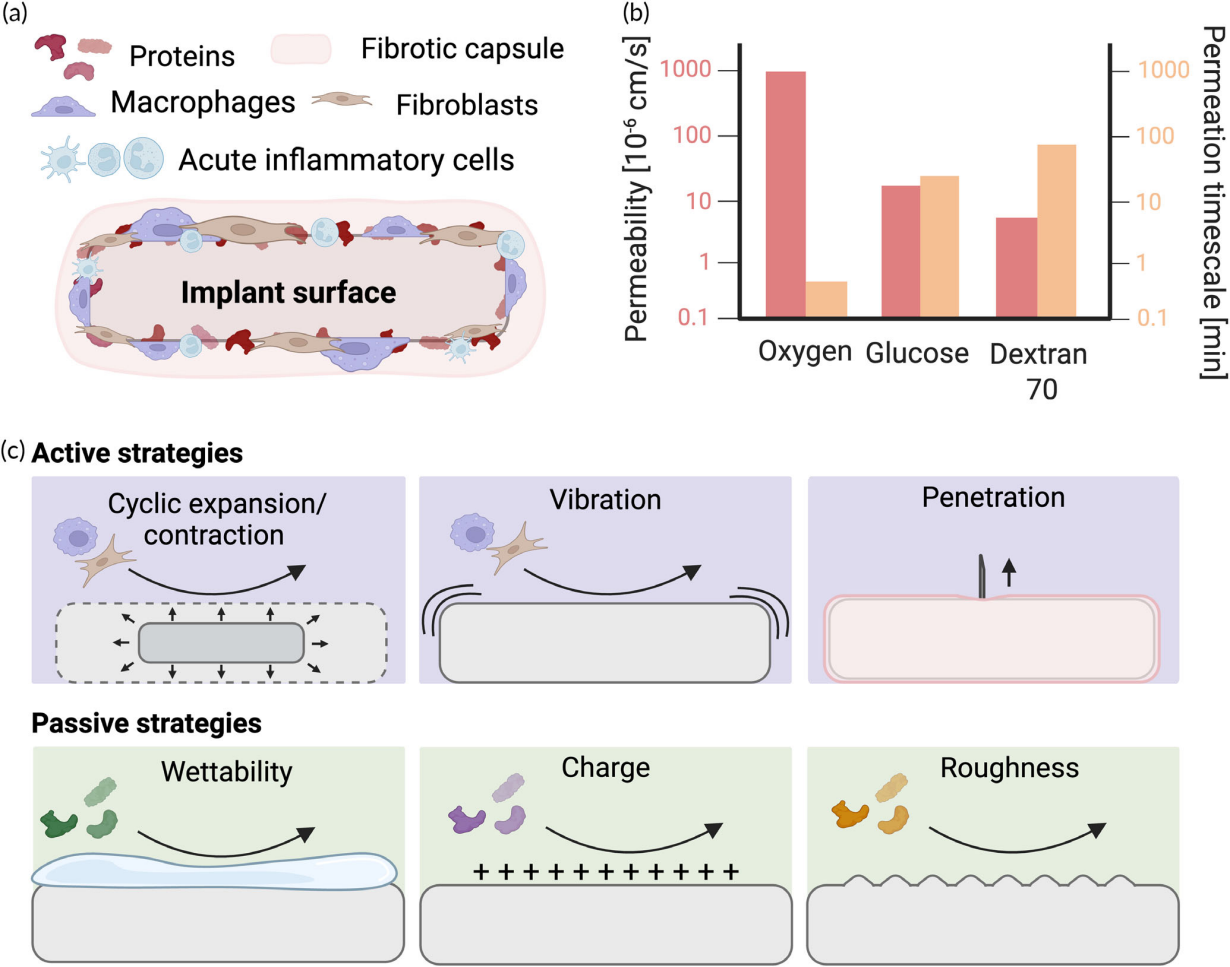
Overview of the foreign body response and mechanisms to overcome it. (a) Composition of implant fibrosis. Acute inflammatory cells include neutrophils, eosinophils, and dendritic cells involved in the initial stages of the foreign body response.^14^, ^15^, ^16^ (b) Permeability and permeation timescale of solutes of increasing molecular weight (Oxygen—32 Da,^17^ Glucose – 180 Da,^5^ Dextran 70–70 kDa^18^) through a 500 μm thick fibrotic capsule. (c) Mechanisms of action to mitigate fibrosis. Created with BioRender.com.

The foreign body response begins immediately upon implantation as proteins from the host encounter the biomaterial (Figure 1a). Blood plasma proteins readily adsorb onto the implant surface and create a provisional matrix with which neutrophils interact within minutes. Within hours, neutrophils recruit macrophages to the site where they bind to adsorbed proteins and spread across the implant surface in an attempt to degrade it. If degradation attempts fail after several days, macrophages recruit fibroblasts that deposit collagen and other extracellular matrix components around the implant. This matures into dense, fibrotic tissue within 2–4 weeks that encapsulates and ultimately isolates the implant from the host.^1^ Implant fibrosis, driven by an accumulation of tightly packed collagen,^19^ often reaches 1–2 mm in thickness.^20^ This collagenous composition is especially detrimental to diffusive mass transport, contributing to decreased permeabilities and increased permeation times of high molecular weight solutes (Figure 1b). Below, we discuss passive and active strategies to target various stages of the foreign body response and ultimately combat its effects (Figure 1c).

## 3 PASSIVE ANTIFIBROTIC STRATEGIES

**FIGURE 2:**
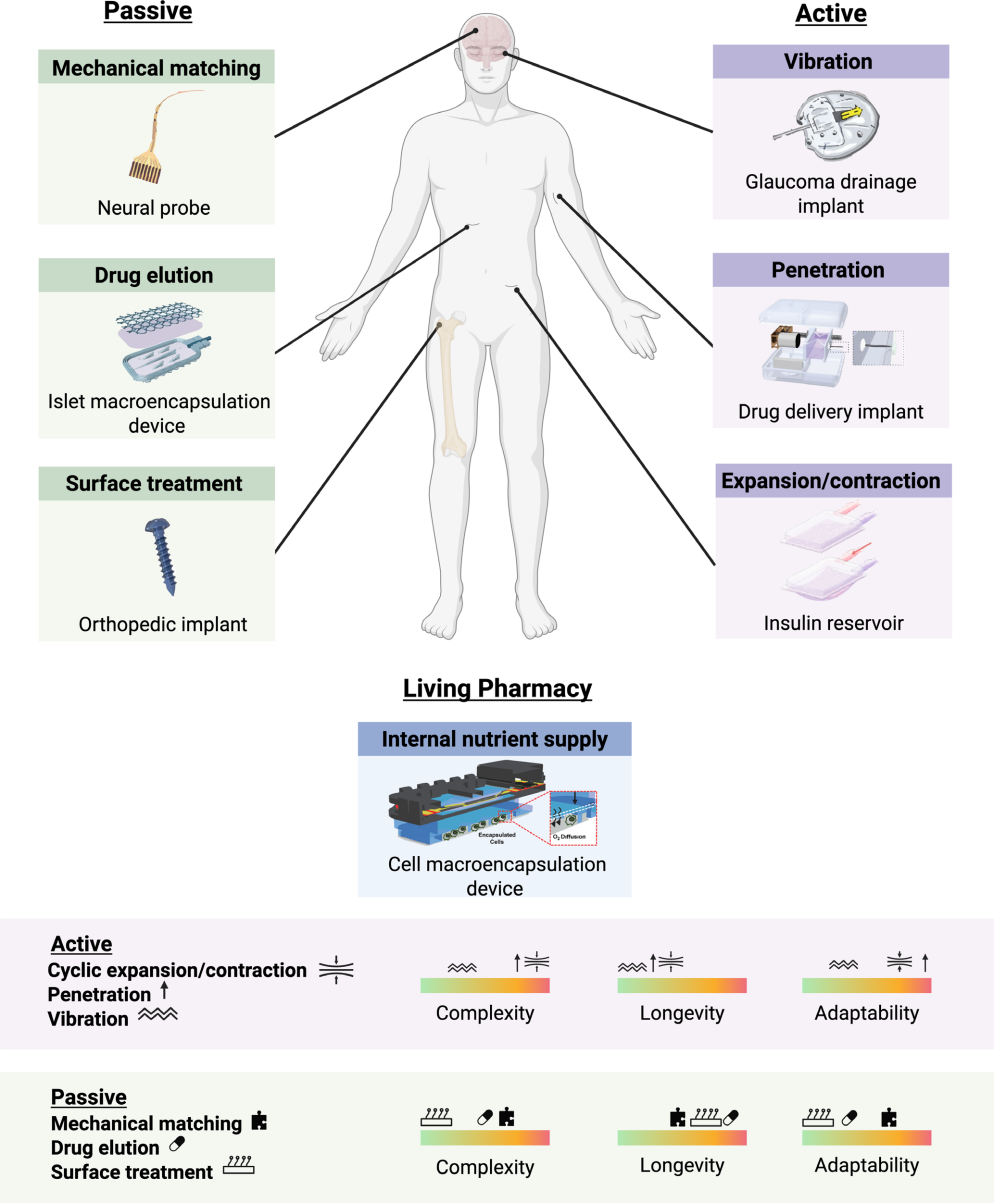
Existing passive and active antifibrotic strategies. (Left) Three categories of passive strategies are highlighted that include an ultraflexible neural probe,^21^ a VEGF‐releasing islet macroencapsulation device,^22^ and a titanium screw with a bioactive‐coating.^23^ (Right) Three categories of active strategies are highlighted that include a magnetoelastic vibrating glaucoma drainage implant,^24^ a needle‐based drug delivery implant,^25^ and a cyclically actuating insulin reservoir.^26^ (Middle) An oxygen‐generating cell macroencapsulation device^27^ is highlighted as an electronic living pharmacy implant. (Bottom) Comparison of complexity, longevity, and adaptability between passive and active strategies.

Passive anti‐fibrotic strategies (Figure 2) rely on superficial or structural implant modifications to mitigate the foreign body response. Superficial modifications directly influence the immune response by dictating the quantity and makeup of proteins that adsorb to the biomaterial surface. For example, decreasing surface roughness generally provides less surface area for proteins to bind and interact with cells, eliciting a weaker response.^11^ Surface wettability and charge both govern protein attraction as well; hydrophilic surfaces tend to discourage protein adsorption due to competition with water molecules, while surfaces and proteins with like charges repel each other.^11^ Controlling these protein interactions can ultimately help implants navigate both the acute and chronic immune response. Structural modifications that mechanically match implant properties with surrounding tissue also improve biocompatibility. For example, matching the elastic modulus of implants with the modulus of surrounding tissue decreases macrophage and fibroblast proliferation.^3^, ^28^, ^29^ These passive antifibrotic mechanisms have been met with success, but the material dependency of these mechanisms may hinder consistent therapeutic efficacy.^29^

Metals, ceramics, polymers, and hydrogels are common implantable biomaterials, each with distinct properties that influence proteins and immune cells differently; as such, the anti‐fibrotic efficacy of passive strategies often depends on the material, complicating the search for a “one‐size‐fits‐all” solution. In mice, cell deposition has been shown to decrease as implant diameter increases from 0.5 to 2.0 mm across six biomaterials (SLG20 alginate, LF10/60 alginate, stainless steel, glass, polycaprolactone, polystyrene).^15^ Other studies with different materials have found the opposite effect. For example, researchers examined the foreign body response in rats to 0.3‐ and 2.0‐mm polyurethane implants and reported that capsular thickness instead increased with implant size.^30^ Another group developed poly(lactic‐co‐glycolic) acid (PLGA)/polyvinyl alcohol (PVA) hydrogel‐coated biosensors of three different sizes (0.3 × 0.3 × 3 mm^3^; 0.5 × 0.5 × 5 mm^3^; 0.75 × 0.75 × 9 mm^3^) and similarly found capsular thickness increased with implant width.^31^ Researchers also report contradictory findings on the influence of surface roughness for different materials. A study revealed 4 μm to be the optimal surface roughness for minimizing the foreign body response around silicone implants in rabbits; deviating topographies induced higher expression of inflammatory cytokines.^32^ A different study, however, showed that adding surface roughnesses of 0.1–18‐μm to polyurethane substrates had no impact on macrophage morphology or immune cell expression of inflammatory markers.^33^ Researchers can optimize implant properties to inhibit fibrosis, but these optimal characteristics vary depending on the material of the device.

When optimized for a specific material, passive strategies, such as immunomodulatory drug‐eluting coatings, can achieve longer antifibrotic mitigation, which is critical for implantable systems that manage chronic conditions. In the case of diabetes, tracking blood glucose with continuous glucose monitors mitigates the risk of sudden, life‐threatening complications. The foreign body response, however, can diminish sensor quality in as early as 3 days.^6^ With drug‐eluting coatings, researchers have extended this functional window from days to weeks. One group, for instance, added a dexamethasone‐eluting coating to Medtronic's MiniMed SOF‐SENSOR™ and reported consistent sensor sensitivity throughout a 3‐week study in rats.^34^ Transplanted islet cells also benefit from drug‐eluting compounds via reduced immune system targeting. One group developed a crystallized, anti‐fibrotic drug that targets a macrophage surface receptor responsible for changes in cell phenotype. The drug reduced fibrosis for 15 months in mice and improved encapsulated islet cell viability compared to uncoated capsules.^35^

Still, drug‐eluting compounds have a finite therapeutic window that depends on drug loading efficiency and release rate; the foreign body response is free to resume once the drug is depleted.^36^ These release characteristics are predetermined and remain unchanged throughout implantation; yet, unpredictable factors in the implant microenvironment can exacerbate the foreign body response at any point during the implant's lifetime. Bacterial infections, for example, add a level of complexity to the biotic‐abiotic interface that influences tissue remodeling and increases fibrosis.^37^, ^38^ Also, erratic movement between the implant and surrounding tissue generates frictional shear stress that heightens pro‐inflammatory cytokine expression, fibroblast activation, and disorderly collagen deposition.^39^, ^40^, ^41^ These frictional forces can arise from walking, running, sleeping, and even breathing.^42^ Interestingly, when applied in a controlled manner, these forces can attenuate fibrotic behavior, forming the basis for active antifibrotic strategies.

## 4 ACTIVE ANTIFIBROTIC STRATEGIES

Active antifibrotic strategies (Figure 2) use dynamic, mechanical stimulation to mitigate the foreign body response by leveraging the effects of controlled, externally induced stress on the cellular environment.^25^, ^43^, ^44^, ^45^ For myofibroblasts, this stress reduces adhesion, proliferation, size, differentiation, and collagen deposition.^46^, ^47^, ^48^ Similarly, cyclic compressive stimulation promotes clearance of neutrophils and factors responsible for neutrophil chemotaxis.^49^ Engineers can capitalize on this behavior by incorporating triggerable mechanical stimuli into implantable devices to directly control cellular dynamics at the implant surface and increase the longevity of the antifibrotic effects. In one study, researchers implemented cyclical compressions into an implantable insulin reservoir using pneumatic actuation. The timing of insulin delivery is critical for maintaining glycemic control, but fibrotic encapsulation inhibits release from implantable pumps. When actuated twice daily for 8 weeks in rats, the device reduced myofibroblast volume and achieved a three‐times greater drop in blood glucose levels compared to a non‐actuating control.^26^ Similarly, researchers developed an engineered hydrogel membrane for implantable glucose sensors that swells and shrinks in response to body temperature fluctuations. This hydrogel promoted cell detachment from the membrane's surface in vitro and reduced fibroblast presence over 90 days compared to a non‐thermoresponsive control in rats.^43^, ^50^, ^51^ Vibration also yields anti‐inflammatory and anti‐fibrotic effects made tunable by modulating the amplitude and duration of actuation.^46^, ^52^ One group developed vibrating magnetoelastic actuators to limit fibrotic encapsulation around glaucoma drainage implants. These implants are meant to reduce intraocular pressure by draining fluid buildup from the eye, but fibrosis increases resistance to fluid flow and causes 10% of drainage devices to fail per year.^53^ The group's device, integrated with vibrating actuators, successfully operated in excised porcine eyes at frequencies shown to disrupt fibroblast adhesion.^24^, ^54^, ^55^ These active mechanisms offer both on‐demand, tunable actuation and sustainable anti‐fibrotic efficacy that passive systems lack.

On‐demand actuation can be coupled with fibrotic‐sensing components that track cellular behavior near the implant surface and relay physiological cues throughout foreign body response progression. Researchers, for example, have used implantable microdialysis probes to sample chemical messengers and gauge fibrotic progression.^56^, ^57^, ^58^, ^59^ When combined with actuators, these integrated systems establish a feedback loop to automatically adjust actuation based on detected fibrotic severity. One study found that tissue remodeling on electrode surfaces induces electrical resistance changes.^60^ Using this principle, researchers engineered a conductive membrane for a cyclically actuating drug reservoir. Algorithms correlated to membrane resistances and capsular volumes adjusted actuation rates accordingly to enable consistent drug delivery.^61^ Another study found that subjecting magnetoelastic materials to vibration generates a magnetic field that varies inversely with cellular mass. Upon actuating magnetoelastic‐coated implants in mice, researchers reported an increased magnetic signal due to a decrease in cell presence, enabling real‐time fibrotic monitoring.^44^ While passive devices are limited by their static nature, devices with active antifibrotic mechanisms can adapt to their dynamic microenvironment and provide informed control over the foreign body response.

Other antifibrotic efforts focus on mechanically perturbing the fibrotic capsule rather than attenuating its formation. While attenuating the capsule permits diffusion of small molecules, macromolecule transport remains challenging.^62^ Bypassing this diffusion barrier altogether by puncturing capsular tissue makes delivery more feasible. In the case of encapsulated glaucoma drainage implants, for example, surgeons restore implant function by disrupting excess scar tissue blocking drainage tubes.^63^ While ad hoc measures by surgeons are useful, directly incorporating mechanisms to puncture fibrosis into the implant diminishes the need for repeated doctor's visits and invasive procedures for implants in deeper anatomical locations. Implantable injection‐pump‐based devices in particular can disrupt fibrosis, and thereby facilitate transport of high molecular weight solutes, such as proteins and nucleic acids, that are otherwise slowed by capsular tissue.^62^ Depending on the injection force, the fibrotic capsule can rupture and give way for the drug to reach its intended target. For example, one group actuated an implantable jet‐injection pump for drug delivery in rats and found that the outlet remained unobstructed 30 days post‐implantation, despite encapsulation on the implant sides.^45^ Another group incorporated a retractable, motor‐driven needle into their implant to pierce through fibrotic tissue whilst delivering a drug bolus.^25^ The implant delivers naloxone to treat opioid overdose, which can become life‐threatening within minutes. As such, it is important that the diffusional barrier of fibrosis is overcome so as not to slow drug transport. Compared to diffusion‐based, bioelectronic devices that take 2 h to achieve 99% drug release,^64^ this convection‐based device achieves complete drug delivery in 25 s. Similarly, engineers used needles to penetrate through a fibrotic capsule surrounding a refillable drug delivery implant 3 months post‐implantation.^65^ Analogously designed needle‐, jet injection‐, and pump‐based devices achieving higher forces penetrate barriers such as the dermis^66^ and gastrointestinal wall,^67^, ^68^, ^69^ so it is crucial that actuation mechanisms for penetrating fibrosis are optimized to avoid damaging surrounding tissue. Additionally, incorporating these active mechanisms necessitates consideration of size constraints and power consumption.

## 5 LIVING PHARMACY IMPLANTS

Circumventing the effects of the foreign body response from within an implantable device, rather than at the tissue‐implant interface, through onboard electronics provides an alternative method to confronting the effects of fibrotic encapsulation, especially for cell‐based therapeutic implants.^70^ These “living pharmacy” implants employ transplanted cells to treat specific diseases, such as pancreatic islet cells for type 1 diabetes, by producing critical biologics.^71^ Transplanted cells rely on nutrients such as oxygen and glucose from surrounding tissue to carry out their designated functions, but fibrotic encapsulation restricts transport.^72^ While applying antifibrotic strategies to the surface of devices that house transplanted cells is an option,^72^, ^73^, ^74^ engineers also developed systems that generate nutrients to supply cells internally.^27^, ^75^, ^76^, ^77^ For example, wireless, battery‐free devices housing islet cells can generate their own oxygen using water electrolysis. When tested for a month in diabetic mice, devices with low oxygen supplies hindered by fibrotic encapsulation showed reduced cell viability that was largely recovered when adding an oxygen‐generating component to the encapsulated implant. Mice possessing the oxygen‐generating implant exhibited greater blood glucose control, with an area under the curve (AUC) under half that of mice with non‐oxygen generating controls.^27^ Another group developed a similar oxygen‐generating device that maintained 73.7% cell viability after 10 days compared to a control which maintained only 26.6% viability.^77^ “Living pharmacy” implants generate nutrients to support cells from within but still must facilitate transport of cell‐made biologics out of the implant through fibrotic tissue. While these devices have shown promise in mice, it will be critical to explore efficacy in larger animal models with more severe foreign body responses^78^ that could prohibit macromolecule drug elution. In the future, these devices could generate nutrients beyond oxygen to further support cell viability from fibrotic encapsulation.

## 6 POWER AND SIZE CONSIDERATIONS

### 6.1 Size considerations

Antifibrotic solutions must conform to site‐specific volume constraints that limit implant size (Table 1). While passive strategies such as modifying surface charge and wettability add negligible bulk material, active strategies often necessitate sizeable integrated or external components such as centimeter‐scale pumps, motors, electronics, and antennae (Table 2). Actuation with pneumatic pumps, for example, requires control systems with an air supply, power source, valves, tubing, and pressure regulators to control flow^81^; adding a solenoid valve alone can nearly double the volume of a human‐scale implantable device.^82^ For needle‐driven devices, integrated printed circuit boards often dictate device length and width, while the motor and battery dictate height. Compared to these components, the onboard microcontroller unit and motor driver chip negligibly contribute to overall size. Incorporating stimuli‐responsive materials such as cyclic expansion/contraction‐ and vibration‐based materials is an alternative active approach to reduce size. For example, wirelessly activated magnetoelastic films with nanometer to micron thickness facilitate integration with implants in confined anatomical spaces such as the eye.^24^ Accompanying external actuation coils which generate the desired magnetic field for antifibrotic effects are still comparatively large though, on the scale of 3 cm in diameter.^44^ Ultimately, minimizing the electronics footprint of active strategies is critical for widespread adoption, as it dictates the invasiveness of the implantation procedure. Importantly, while smaller implants tend to elicit a weaker foreign body response, mechanical actuation protocols can be tuned accordingly^61^ if a larger implant size is non‐negotiable.

**TABLE 1:** Implant sizes in different anatomical locations.

Location	Implant type	Name	Volume [mm^3^]
Eye	Subretinal prosthetic implant	PRIMA	0.12^79^
Eye	Intraocular drug delivery implant	Ozurdex	0.38
Eye	Intraocular drug delivery implant	Yutiq	0.997
Brain/skull	Deep brain stimulation electrode	SenSight	11.4
Brain/skull	Microelectrode array	Utah Array	24
Brain/skull	Neuromodulator	NeuroPace RNS	11,000
Subcutaneous	Cardiac monitor	Assert‐IQ	1900
Subcutaneous space	Defibrillator	EMBLEM	59,500
Subcutaneous space	Infusion pump	SynchroMed	114,562

**TABLE 2:** Dimensions of components found in active devices.

Component	Smallest dimension [mm]	Largest dimension [mm]	Power consumption (W)
Motor driver chip^25^	0.8	2.1	15 × 10^−9^ – 4.5 × 10^−3^ (at 3 V)
Microcontroller unit^25^	0.88	5	300 × 10^−9^ – 29 × 10^−3^ (at 3 V)
Pump^80^	12	30	0.12
Solenoid valve^26^	18.5	67.8	2–4
Battery^80^	7	60	‐

### 6.2 Power considerations

Many active strategies require either batteries or antennae to power electronics that enable mechanical stimulation and feedback control, increasing the size, safety, and cost of devices.^83^ Lithium‐based batteries (Table 3) offer low discharge rates and high energy densities but pose risks due to their toxicity and flammability.^84^ Silver oxide batteries pose comparatively lower toxicity risks and are less susceptible to overheating, making them a safer but less energy‐dense powering option.^84^ Ultimately, all batteries introduce a level of concern unique to active strategies but are often necessary for powering onboard electronics.^85^ For devices that purely perform mechanical stimulation on fibrotic tissue, power consumption depends on the chosen microcontroller (MCU), actuator, and actuation regimen. Previous devices have used a low‐power ATmega328P MCU and miniature peristaltic pump with max current draws at 3 V of 1.5 and 40 mA, respectively.^80^ With 5‐min actuation every 12 hs, the system's Li‐polymer 803,860, 2000 mAh battery could last more than 6 months before needing to recharge. However, this battery is 18.24 cm^3^ in volume; an implantable 1.2 cm^3^ Li‐polymer 70 mAh battery would last less than 2 weeks, and two 0.15 cm^3^ 40 mAh silver oxide batteries in series would last less than 1 week. In addition to devices that require repeated actuation, some devices only require one‐time burst release administration to penetrate fibrotic tissue, proportionally reducing their onboard power requirements.

**TABLE 3:** Summary of common implantable batteries.

Battery	Open circuit voltage [V]	Energy density [Wh/kg]
Silver oxide	1.6	150–250^84^
Lithium iodine	2.8	210–270^86^
Lithium manganese oxide	3.3	230–270^86^

In closed‐loop systems, an added sensor can consume around 10 times as much current as the MCU itself^25^, ^87^; however, depending on the actuation mechanism in the system and the number of times the implant requires actuation, the sensor's current draw may be negligible. The device designed to provide a single burst release of naloxone following an opioid overdose incorporates a motor‐driven pump with a 35 mA free‐run current draw, 30 times greater than its sensor with the highest current draw.^87^ Similar devices for preventing opioid overdose with pumps drawing 50 mA dwarfed the 1.4 mA onboard sensor current requirements.^25^ However, because pumps that deliver a burst release only need to run once, this power consumption is within the batteries' capacity and does not pose a significant limitation on device operation. Sensors, on the other hand, must run continuously; a sensor operating at 3 V with a maximum current draw of 1.5 mA would consume approximately 363 J per 24 h of on‐time, while a pump drawing 50 mA for a single 25‐s actuation event consumes 3.75 J total. Given the continuous energy demands of sensors and the large energy demands of pumps, intermittent or one‐time actuation events are necessary for optimizing device longevity in active systems.

Compared to batteries, wireless power transfer offers an unlimited power supply but suffers from large external antennae, signal attenuation at increased depths, and constrained current draws, limiting its applicability to superficial implants.^88^ Stimuli‐responsive materials that undergo physical changes in response to environmental factors, such as magneto‐responsive hydrogels, offer battery‐free actuation without external power, though manual operation is required.^89^ Ultimately, active solutions may prevent fibrosis for longer, but most need constant power and have limited battery lives. Therefore, this mechanical approach is best suited for devices that have long residence times in the body with limited numbers of actuation events.

## 7 OUTLOOK AND CONCLUSION

**FIGURE 3:**
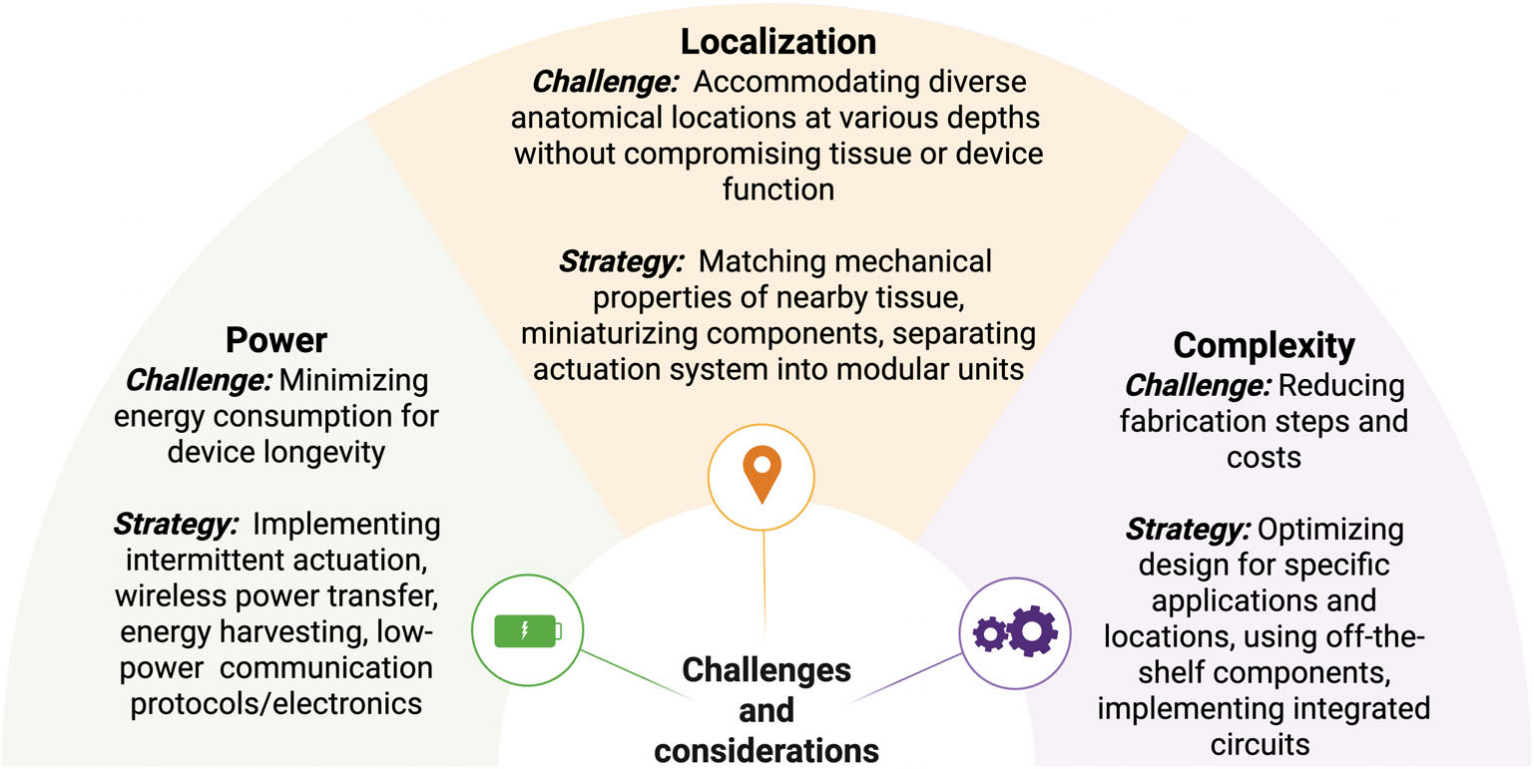
Challenges and considerations for progressing the development of active antifibrotic strategies. Created with BioRender.com.

Antifibrotic strategies that incorporate mechanical stimulation such as vibration or cyclic expansion into implantable devices offer a long‐lasting approach to overcoming the foreign body response in a variety of implantable devices, including drug delivery systems, biosensors, prosthetics, and neural probes. While passive strategies such as coatings are cheaper and more space‐efficient due to their lack of electronic components, they exhibit material‐dependent efficacy and cannot adapt to the foreign body response over months‐long timescales. Conversely, active strategies offer tunable, on‐demand actuation and facilitate integration with fibrotic‐sensing components across materials; however, excessive power consumption of integrated electronic components and limited battery space make these strategies best for long‐lasting systems that require only intermittent use (Figure 3). Active strategies have mitigated fibrotic encapsulation of implantable drug delivery systems and biosensors, but the extension of these strategies to other implantable devices such as pacemakers and prosthetic implants remains largely unexplored. These applications will require unique solutions to accommodate diverse anatomical locations without compromising tissue or device function. Active antifibrotic strategies have shown promise in the eye, subcutaneous tissue, and the brain, but future work needs to investigate efficacy in other common implant sites such as the teeth, bone, and ears. Modularization, miniaturization, and mechanical matching to nearby tissue could make active strategies more versatile, minimize surgical complexity, and reduce patient discomfort. For devices designed to rupture fibrotic encapsulation, characterizing biomechanical properties could furthermore help minimize the power required to operate and minimize damage to surrounding tissue. The differences between utilizing these strategies in animal models and humans also remain largely unexplored. While mechanical actuation persists as long as external stimuli or battery power are supplied, translating from animal models to humans will necessitate tuned actuation protocols. Though devices utilizing needle penetration to overcome fibrotic strategies have been tested in large animal models, cyclically expandable hydrogels, magnetoelastic vibrating implants, and jet injection pumps still require further testing to ensure similar efficacy in large animals. These actuation methods were tested for up to 90 days in vivo, but further analysis at longer timepoints could provide additional evidence to support long‐term efficacy. Further analysis should also be performed by characterizing what happens to displaced collagen and other ECM components following mechanical perturbation. Continued advancements are poised to enhance the functionality and lifespan of implantable medical devices, leading to improved patient outcomes.

## AUTHOR CONTRIBUTIONS

J.J. and A.A. conceptualized and wrote the manuscript. L.T. assisted in writing the manuscript.

## CONFLICT OF INTEREST

A.A. is a co‐inventor on ingestible devices for drug delivery. A.A. is a consultant for Novo Nordisk on ingestible drug‐delivery devices. A full list of A.A.'s competing interests can be found here: https://www.abramsonlab.com/aa-conflicts-of-interest.

## Data Availability

Data sharing is not applicable to this article as no new data were created or analyzed in this study.
